# Household-Level Spatiotemporal Patterns of Incidence of Cholera, Haiti, 2011

**DOI:** 10.3201/eid2009.131882

**Published:** 2014-09

**Authors:** Jason K. Blackburn, Ulrica Diamond, Ian T. Kracalik, Jocelyn Widmer, Will Brown, B. David Morrissey, Kathleen A. Alexander, Andrew J. Curtis, Afsar Ali, J. Glenn Morris

**Affiliations:** University of Florida, Gainvesille, Florida, USA (J.K. Blackburn, U. Diamond, I.T. Kracalik, A. Ali, J.G. Morris Jr);; Virginia Polytechnical Institute and State University, Blacksburg, Virginia USA (J. Widmer, K.A. Alexander);; Sustainable Aid Supporting Haiti, London, United Kingdom (W. Brown);; FISH Ministries at Christianville Foundation, Inc, Gressier, Haiti (B.D. Morrissey);; Kent State University, Kent, Ohio, USA (A.J. Curtis)

**Keywords:** Cholera, bacteria, Vibrio cholerae, Haiti, spatiotemporal, SaTScan, GIS, GPS, foodborne, waterborne, diarrhea

## Abstract

A cholera outbreak began in Haiti during October, 2010. Spatiotemporal patterns of household-level cholera in Ouest Department showed that the initial clusters tended to follow major roadways; subsequent clusters occurred further inland. Our data highlight transmission pathway complexities and the need for case and household-level analysis to understand disease spread and optimize interventions.

The 2010–2011 Haiti cholera epidemic was one of the largest worldwide in recent history. Before the initial outbreak, cholera had not been reported in Haiti for at least 100 years (*1*). Multiple factors likely contributed to the magnitude and spread of the early outbreak, including lack of prior exposure to cholera among the population, genetic characteristics of the *Vibrio cholerae* strain, and the consequences of the January 2010 earthquake, which included mass destruction of the infrastructure of Haiti and displacement of 1.5 million persons. The water and sanitation infrastructure in Haiti were inadequate before the 2010 earthquake; much of the population had no access to treated drinking water (48%) or sanitation facilities (75%) (*2*). Many of the limited services were destroyed by the 2010 quake (*3*).

As of February 28, 2013, the Ministry of Public Health and Population (MSPP) of Haiti and the National Directorate for Water Supply and Sanitation, working with the Pan American Health Organization, announced an ambitious plan to eradicate cholera from Haiti and Hispaniola: the plan calls for aggressive efforts to improve sanitation and to vaccinate the entire Haitian population (*4*). Designing this plan for optimal operation requires an understanding of cholera transmission within the population, and the development of models that permit assessment of the impact of proposed interventions on disease incidence. However, epidemiologic studies on the Haiti cholera outbreak have focused on the diffusion of the disease by using aggregated data, such as those from arrondissements (*5*) and communes (*6*). To evaluate cholera case clustering and provide a basis for modeling and intervention design, we conducted a spatiotemporal analysis of household-level data in the Ouest Department in Haiti during 2010–2011.

## The Study

**Figure 1:**
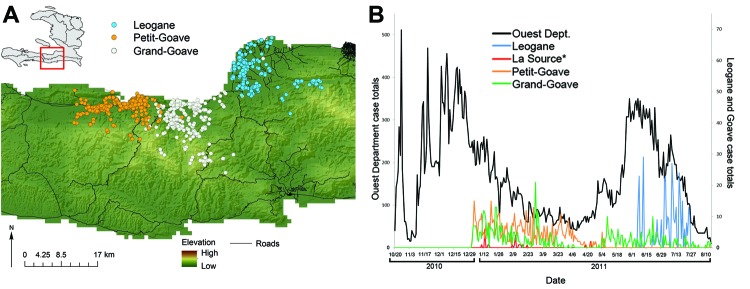
Findings of the Cooperative Cholera Mapping Project in Haiti, 2011. A) Geographic distribution of household cholera cases per day. B) Temporal pattern, color-coded by community, compared with reported cases in the Ouest Department (black). Color coding of map symbols in A correspond to line colors in B. La Source cases (n = 25) are plotted but not mapped.

We used data from the Collaborative Cholera Mapping Project (CCMP), a Web-based dataset (no longer online) of household-level cholera cases captured through the United Nations Children’s Fund water, sanitation, and hygiene program in the Leogane/Petit Goave area and Sustainable Aid Supporting Haiti (https://www.facebook.com/SASHaiti/info), a private nongovernmental organization (NGO) working in the area. The CCMP contains case data from 4 communities that include 3 urban areas, Petit Goave, Grand Goave, and Leogane (Figure 1, panel A). The fourth community in the CCMP is La Source, a small community in the west on Highway 7 (not mapped). Case data are summarized in the Table; full details on the urban structure of each community are provided in the Detailed Description of the Four Communities in the Collaborative Cholera Mapping Project (Technical Appendix).

The CCMP compiled global positioning system (GPS) data on household locations of patients who reported to cholera treatment centers during January–August 2011. Households were visited by community health workers employed by NGOs in the region. Persons in each household were educated about cholera, and surfaces in the house were disinfected. The GPS coordinates were entered into the dataset by NGO personnel.

Data were provided by CCMP with exemption from the University of Florida Institutional Review Board. Participants were not identified. We constructed a geographic information system, or GIS, database of household cases by date for each of the 3 communities on Route 2 and evaluated them separately. We plotted CCMP cases against daily case incidence reported to MSPP for the Ouest Department to evaluate the temporality of CCMP data.

We evaluated space-time clustering of cases for each community by season (winter or summer), using the spatial scan statistical tool in SaTScan (http://www.satscan.org). We used the retrospective space-time permutation model (*7*), which does not require data for population at risk, a key factor given the lack of reliable post-earthquake population data for Haiti (online Technical Appendix, Geospatial Analyses). Households were used as case locations. Clusters were identified as a maximum cluster size of 50% of case data and a maximum temporal window of 50% of the study period (Technical Appendix, Geospatial Analyses).

There was close agreement in the trend of daily CCMP and MSPP data (Figure 1, panel B), illustrating 2 seasonal peaks for the epidemic, with fewer total cases in the dryer winter months and very close agreement in the timing of seasonal peaks between CCMP and MSPP cases. Datasets were not complete for all time periods for all sites (Table), reflecting some element of bias in identification of households related to operational factors such as availability of personnel to follow-up cases and enter case data into the dataset. Nonetheless, there was a clear pattern of disease movement from CCMP data, with winter/spring cases seen in La Source, Petit Goave, and Grand Goave, followed by ongoing summer cases in Grand Goave, and movement of the epidemic eastward into Leogane, with some early spring cases in Leogane.

**Table:** Case count and date ranges of cholera case reports by community, Haiti, 2011

Community	Total no. cases	Date range of cases (no.)
Petit Goave	612	Jan 1–May 6 (612)
Grand Goave	549	Jan 1–Apr 7 (348); May 1–Aug 15 (201)
Leogane*	344	Feb 7–May 7 (61); Jun 7–Jul 26 (283)
La Source	25	Jan 10–Feb 22 (23); Apr 23 (2)

*Cases in Leogane were few for the reporting period of Feb 7–May 7; related data were not included in further space-time analysis.

**Figure 2:**
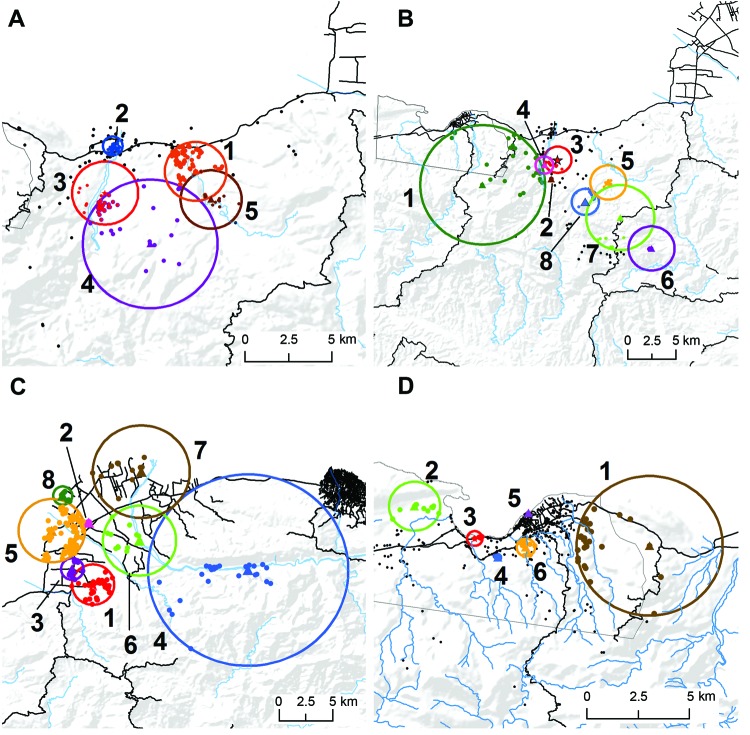
Space-time clusters of cholera in 4 communities in Haiti, 2011. A) Grand Goave, winter; B) Grand Goave, summer; C) Leogane, summer; and D) Petit Goave, summer. Stars represent primary cluster centers and triangles, secondary cluster centers. Dots represent approximate locations of households within clusters. Clusters are numbered sequentially by order of date of occurrence.

In Grand Goave, long lasting (≈20 days) winter case clusters appeared in early January at the confluence of a natural waterway and Route 2, the major highway into the southern peninsula (Figure 2, panel A). This was followed by springtime cases further inland/upland in rural areas and along waterways (Figure 2, panel B). Summer clusters that followed were in rural, inland/upland areas, with no clusters identified in the urban center. In Leogane (summer), clusters were again identified along Route 2, beginning south of the city and in the urban center (Figure 2, panel C). Later clusters appeared the mountains, in communities along the Momance River, with subsequent clusters following the river toward the Caribbean Sea. Four of 6 clusters in Petit Goave were situated between 2 major highways with 2 clusters near the urban center (clusters 5, 6) (Figure 2, panel D). The first 2 clusters appeared east (cluster 1) and west (cluster 2) of the urban center in semirural regions along natural waterways.

## Conclusions

Here we provide an initial spatiotemporal assessment of household-level cholera following the introduction of *V. cholerae* to Haiti. Our 2011 winter/spring cases occurred in the initial larger epidemic wave, followed by additional peaks in cases in summer, with the onset of the rainy season. Our data support the hypothesis that initial case transmission followed roadways, particularly Route 2. In Petit and Grand Goave, transmission along roadways was followed by disease movement into rural/inland areas. After initial urban case clusters occurred in Leogane, cases appeared in the mountains, with clusters then appearing along the Momance River, consistent with the hypothesis that the river provided a transmission route for *V. cholerae*. Supporting this observation, toxigenic *V. cholerae* O1 was recently recovered from the Momance River (*8*).

There is increasing recognition that cholera has 2 routes of transmission, one involving movement through waterways (e.g., surface waters, rivers) and the other related to more direct transmission from person to person (*9*,*10*). In keeping with recent mathematical models (*11*), our data support the hypothesis that both routes are important to transmission in Haiti. The inland/river movement in both Petit Goave and Leogane occurred during the summer rainy season, consistent with a link between transmission involving surface waters and seasonal rainfall. These data were collected early in the course of the epidemic and limited to a small proportion of the total reported cases. Multiple years of observation are necessary to confirm these patterns; however, models in this and other regions already suggest the development of a seasonal pattern of illness linked with rainfall (*12*).

Generally, human mobility, such as urban/rural or rural/urban migrations can influence disease patterns (*13*). Our results suggest that such mobility is a factor of epidemic cholera transmission in Haiti. Recent models suggested human movevent out of damaged areas was substantial, but ultimately, persons attempt to return to the areas that formed the basis of their predisaster social networks (*14*). This study identifies key geographic areas for improved data collection. It also highlights the need for careful targeting of interventions that are shaped by ongoing data collection and analysis at local levels. Transmission routes can differ across space and time, and only by understanding these local differences can cost-effective disease control methods be identified and implemented.

Technical Appendix Detailed description of the 4 communities in the Collaborative Cholera Mapping Project and geospatial analysis of outbreak clusters.
