# Supplementary material for: Household-Level Spatiotemporal Patterns of Incidence of Cholera, Haiti, 2011
Source: Emerg Infect Dis. 2014 Sep;20(9):1516–9. doi: 10.3201/eid2009.131882 (PMC4178390; doi:10.3201/eid2009.131882)
Supplement: Technical Appendix — Detailed description of the 4 communities in the Collaborative Cholera Mapping Project and geospatial analysis of outbreak clusters. [file 13-1882-Techapp-s1.pdf]

DOI: http://dx.doi.org/10.3201/eid2009.131882

Household-Level Spatiotemporal Patterns of Incidence of Cholera, Haiti, 2011

Technical Appendix

Detailed Description of the Four Communities in the Collaborative Cholera Mapping Project

Petit Goave

The urban center Petit Goave suffered significant damage from the 12 January 2010 earthquake and was subsequently damaged by a 5.9 aftershock which had an epicenter that was almost directly under this urban center on 20 January 2012. This damage was significant on a regional scale, as the urban center of Petit Goave services not only populations in the western portion of Ouest, but also communities significantly west of the urban center along Route Nationale 2. As a result, there was a sizable NGO presence immediately following the 2010 earthquake. That presence has since been reduced to only a few satellite offices of NGOs still working in the Leogane area.

While much of the urban infrastructure was damaged as a result of this seismic activity, there remains a strong urban pattern that continues to define settlement patterns and population densities. The urban pattern is largely confined by a complex surface water system comprised of canals and natural seasonal streams and rivers. There are two dominant urban nodes in Petite Goave, one predominantly dedicated to commercial and civic activities along Route Nationale 2 and spreading northwest toward the formally-planned, historic urban center. The second urban node is dedicated largely to residential land use, where more formal settlement patterns are found adjacent to the commercial urban node and transition into more informal settlement patterns as this residential area moves northwest toward the coast.

Grand Goave

While Grand Goave has a distinct and formally-gridded urban pattern, this urban center is far less populated than Petit Goave, particularly in terms of its urban population. Grand Goave is characterized by a confined urban center with most roads running north toward the coast from

Route Nationale 2. Grand Goave quickly transitions away from a formally-planned urban center toward a more rural area to the south of Route Nationale 2. While Grand Goave is a compact and well-defined urban environment, the urban center does not support a significant residential population. Grand Goave’s housing density is concentrated to the south of the urban center and Route Nationale 2 heading toward the mountains. Of the three urban centers included in the Collaborative Cholera Mapping Project (CCMP), Grand Goave is smallest in population, as well as succumbed to the least amount of damage from the 2010 earthquake.

Leogane

Leogane is a formally-planned, well-confined urban center that lies inland from the coast and northwest of Route Nationale 2. The 2010 earthquake damaged approximately 80–90% of the built environment in Leogane. In response to the significant damage to housing in the area coupled with the lack of basic resources and services in the area, a large concentration of NGOs established relief and rebuilding efforts in the area. Despite the significant and sustained damage to the area following the 2010 earthquake, Leogane continues to be the commercial hub for many communities west of Port-au-Prince, as well as a major thoroughfare for individuals traveling to and from Port-au-Prince to western and southern regions of Haiti.

The urban center of Leogane lies along Route Nationale 2 at the confluence of alluvial plains to the north and mountains to the south. While there is a rapid rural to urban gradient from Route Nationale 2 into the mountains to the south and toward the coast to the north, a lack of roads and transportation services limits the exchange between the rural and urban dynamics, in addition to limiting access to services that are located in the urban center of Leogane. Thus, there is a stark contrast between conditions in the rural outskirts of Leogane and the urban corridor along and to the north of Route Nationale 2, where infrastructure related to water, sanitation, storm drainage and electricity are most robust.

The urban center of Leogane is serviced by a series of ancillary roads that feed into the formal grid pattern of the city. Route Nationale 2 bypasses the formal city center of Leogane. Informal development along the highway (and away from the urban center of Leogane) is the resulting urban phenomenon. This development lies to the south of Leogane and spreads southeast toward smaller urban centers. This is significant as dwelling patterns are also following the urban sprawl away from and to the northeast of Leogane.

One major highway connects the communities represented in the CCMP. Highway Nationale 2 runs from west to east through Petit Goave, Grand Goave and the rural area of Carrefour Fouche to the east of Grand Goave before turning north towards Leogane, continuing on as the major thoroughfare into the capital city of Port-au-Prince.

La Source

La Source is a small community in western Haiti along Highway 7. It sustained heavy damage during the earthquake, with reports of up to 50% of all structures sustaining damage or being destroyed. This community is the western most community in the CCMP and disjunct from the other communities.

Geospatial Analyses

In this study, we were limited to human cholera case data and lacked human population data at a comparable resolution to derive a population at risk for either the Bernoulli or Poisson implementations of the space-time statistics in SaTScan. Given that those cases were recorded at the GPS unit of each household, we had a rare opportunity to map the cholera outbreak at high spatio-temporal resolution. We used the space-time permutation model described in detail by Kulldorff et al. (*1*). Briefly, the space-time permutation model derives case expectation under the assumption that all case dates and locations are independent of any spatial interaction. Next varying sized cylinders are placed over the study area, with the diameter of the circle representing the spatial area scanned and the height of the cylinder representing time. This procedure matches other population-at-risk models available in SaTScan. To determine if case-only data are clustering in space and time, the number of cases in a cylinder is compared to case expectations outside of the cylinder and evaluated using a Poisson generalized linear ratio (GLR). The full mathematics of this are reported in Kulldorff et al. (*1*). Across a large number of cylinders evaluated across the landscape and over the study period, the cylinder that maximizes the GLR is considered the cluster least likely to be random, or the most likely cluster. Additional significant clusters are defined as secondary clusters.

For this study, we used SaTScan v9.1.1 (www.satscan.org) to run the spatial scan statistic. Each case was assigned the geographic location of the household from the CCMP field efforts and the day of the case was determined relative to the first day of reporting in the CCMP.

We set the spatial window at 50% of the household distribution and the time window at 50% of the total days cases were reported. We used the natural break in case reported between the winter time months and later rainy summer months (see Figure 1, panel B in the main text) to divide the study into two time periods (winter/dry, summer/wet). SaTScan models were constructed for each of the three communities, Leogane, Petit Goave, and Grand Goave, separately for each season. Clusters were mapped in ArcGIS v10 (ESRI, Redlands, CA, USA) by mapping cluster centers from SaTScan and placing a buffer around each center using the size of the cluster. Here we considered both primary and secondary, so long as each was statistically significant at $p < 0.05$. SaTScan identifies cluster members (here households) for each clusters. We mapped those and color coded them with the cluster center and buffer. Clusters were identified sequentially by the initial date of cluster appearance.

Reference

1. 7. Kulldorff M, Heffernan R, Hartman J, Assunção R, Mostashari F. A space–time permutation scan statistic for disease outbreak detection. PLoS Med. 2005;2:e59. PubMed http://dx.doi.org/10.1371/journal.pmed.0020059
